# Relationship Between Human Capital and Technological Innovation Growth of Regional Economy and Psychology of New Entrepreneurs in Northeast China

**DOI:** 10.3389/fpsyg.2021.731284

**Published:** 2021-12-02

**Authors:** Xingyang Yu, Mingji Liu

**Affiliations:** ^1^School of Economics and Management, Harbin University of Science and Technology, Harbin, China; ^2^School of Public Finance and Administration, Harbin University of Commerce, Harbin, China

**Keywords:** new entrepreneurs, educational psychology, awareness of innovation and entrepreneurship, human capital, regional economic growth

## Abstract

The economic restructuring and rapid rise of the economy in Northeast China have resulted in a proliferation of new ventures. Studying the psychology of new entrepreneurs is conducive to understanding the relationship between human capital and economic growth. The work reported here aims to explore the impact of human capital on economic growth in Northeast China and the influencing factors of psychological capital of new entrepreneurs in the entrepreneurial process. Based on Cobb–Douglas production function, the relationship between labor, physical capital, or human capital and economic growth in Northeast China is analyzed by econometric methods, and a model of human capital and economic growth in Northeast China is constructed. Besides, a psychological capital intervention (PCI) model is proposed to develop the psychological capital of new entrepreneurs, and the psychological quality structure model of entrepreneurial entrepreneurs and its operation mechanism. The results of the empirical analysis demonstrate that the elasticity coefficient of human capital in Northeast China is 0.15902, five times smaller than that of labor and physical capital. Moreover, 70% of new ventures are willing to accept higher education. The fitting degree of using the PCI model to develop the psychological capital of new ventures is only 0.3%. In addition, the modified external environment PCI instead of the external environment PCI model has a huge operating potential in the macro-entrepreneurial environment. In conclusion, the impact of human capital on economic growth in the northeast is smaller than the impact of labor and material capital investment on regional economic growth. The development of human capital and research on the composition and mechanism of psychological quality of entrepreneurial entrepreneurs are of significant theoretical and practical values to promote the economic growth in the northeast.

## Introduction

Since the reform and opening up, China’s economy has developed rapidly. However, the northeast region has lagged behind other regions of China after the establishment of the planned economic system, and there have also been two “northeast phenomena.” Technology, human capital, and cultural factors are all affecting the economic growth of Northeast China. Tasawar (2019) suggested that, in the context of economic theory, human capital has become a new factor of production after physical capital and labor, which plays a critical role in regional economic growth. Xin and Wang (2019) pointed out that the backward economic environment and arduous living material conditions in the northeast cannot satisfy the talents’ pursuit of high income and superior working environment and living environment, failing to retain talents from within and outside the region, which leads to a great loss of talents. Jia and Gu (2019) stated that Northeast China has strong educational strength and prominent human capital advantages, but the human capital in Northeast China does not play a great economic utility, especially numerous new entrepreneurs belonging to human capital.

There are diversified foreign studies on new entrepreneurs and economic growth currently. Rauhut and Humer (2020) discussed the assumption on how economic growth spreads in space and compared it with different economic theories. They proved that strategic EU documents increasingly promoted the urban dimension and concentrated resources on cities at the expense of regional development cohesion. Fatma et al. (2020) found that psychological factors had a certain effect on business venture success. Especially, entrepreneurial overconfidence and optimistic bias affected new entrepreneurial success to a large extent. Domestic research in this field is still in the stage of recommendation and introduction. Zhang (2019) suggested that individual characteristics and characteristics of entrepreneurs had a vital impact on the international expansion behavior of start-up small- and middle-sized enterprises. However, the existing findings mainly focused on the characteristics of entrepreneurs matching with the high uncertainty and high ambiguity of the overseas market environment. Qi and Meng (2020) indicated that individual subjective factors were the main source of entrepreneurs’ excessive psychological pressure. Moreover, they believed that it is necessary and feasible to explore the cultural path of resolving entrepreneurs’ psychological tension from the perspective of the impact of culture on the individual mental world. Despite many related studies on basic human capital and the effect of human capital on economic growth, the influence of knowledge-based human capital on economic growth is also worthy of attention, which is mainly studied here. Qian et al. (2018) discussed the role of feedback seeking in the relationship between leadership and task performance and the relationship between responsibility and discourse power, based on the social communication theory. Although there are many related works on the role of basic human capital and the effect of human capital in economic growth, attention should also be paid to the impact of intellectual human capital on economic growth. Therefore, the impact of intellectual knowledge human capital on economic growth is primarily studied here.

Here, the relationship between human capital and economic growth and the relationship between economic growth and entrepreneurial psychology of new entrepreneurs in Northeast China are investigated by a combination of questionnaire survey, model construction, and empirical analysis. The innovation of this investigation is to establish a psychological research model of entrepreneurial entrepreneurs based on the Douglas production function. Besides, economic approaches are utilized to analyze the relationship between labor, material capital, or human capital and economic growth in Northeast China, which is suitable for the study of entrepreneurial psychological capital.

## Research Methods

### The Definition of Psychological Quality and Psychological Traits of Entrepreneurs

**FIGURE 1:**
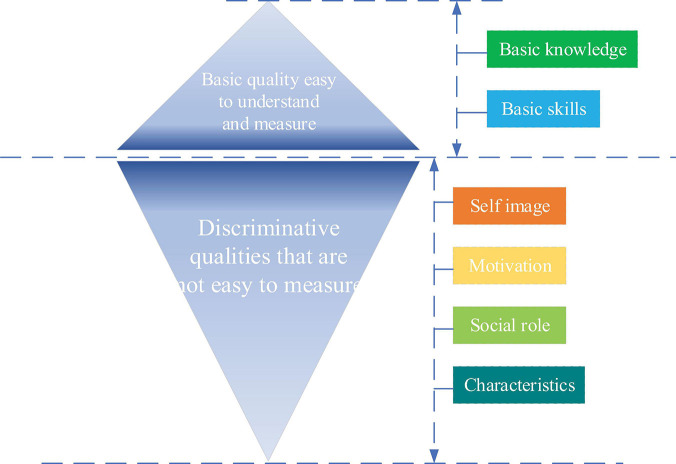
Iceberg model for quality.

The iceberg model for quality takes the help of an iceberg which has just a part of its volume above water and the rest remains beneath the surface in the sea to explain the concept of individual quality. The structure is shown in Figure 1. Similarly, a quality has some components visible like basic knowledge and basic skills, which are easy to understand and measure and relatively easy to change and develop through training. However, other components beneath the surface including social roles, self-image, traits, and motivation are inherent and difficult to measure. Knowledge and skills above water surface can be defined as the benchmark quality. Rogoza et al. (2018) identified components of quality hidden deeply as the differentiating quality, such as the internal drive, social motivation, personality quality, self-image, attitude, self-esteem, narcissism, and self-worth. Related to knowledge and skills, differentiating qualities are not easy to be observed and measured and difficult to change and evaluate.

**FIGURE 2:**
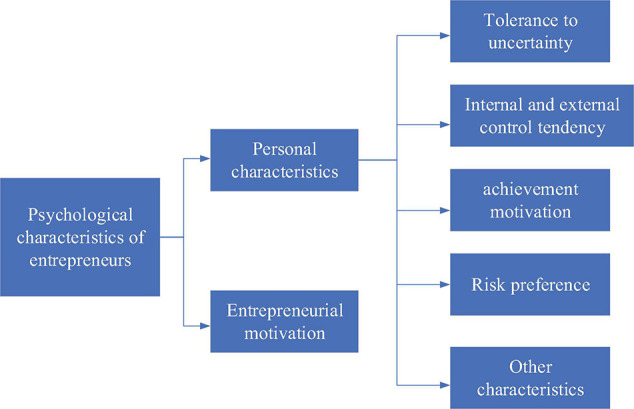
Psychological characteristics of new entrepreneurs.

Jayawickreme et al. (2020) proposed that the theory of personality traits in psychology believed that personality is composed of many traits which refer to the qualities or characteristics of people affecting their behaviors and acting as a general, stable, and lasting behavioral tendency. Moghadam et al. (2020) put forward that these traits are seen as a neuropsychological structure and also a preexisting tendency to make individuals respond to stimuli in a relatively consistent manner, including achievement motivation, internal and external control tendencies, and tolerance for uncertainty. Obschonka et al. (2020) indicated that correspondingly, the influencing factors of psychology of new entrepreneurs can be summed up as awareness of innovation and entrepreneurship, entrepreneurial motivation, entrepreneurial concept, entrepreneurial values, and entrepreneurial ability, which jointly determine the success or failure of new ventures. Psychological characteristics of new entrepreneurs are shown in Figure 2.

### Model Construction of Human Capital and Economic Growth in Northeast China

The adopted Cobb–Douglas production function is generally expressed as Equation 1.


(1) $$Y=A\cdot L^{\alpha }\cdot K^{\beta }$$

In Equation 1, *Y* represents the economic aggregate; *K* denotes the material capital; *L* shows the labor force; and *A* shows the technical level. Besides, *α* and *β* are the output elasticity of labor and material capital, respectively.

It is necessary to introduce the variable of human capital and estimate the output elasticity of human capital to economic growth to study the relationship between human capital and economic growth. Based on this, further comparison of output elasticity is carried out among material capital, labor force, and human capital to regional economic growth. In the proposed model of human capital and economic growth, the introduced variable of human capital to the original model-Douglas production function model is defined as *H*. Then, the improved equation can be written as follows:


(2) $$Y=A\cdot L^{\alpha }\cdot K^{\beta }\cdot H^{\gamma }$$

where *K* is the material capital; *H* represents the human capital; *L* denotes the labor force; and *A* shows the technical level. Moreover, α and β are the output elasticity of labor and material capital, respectively, and γ is the output elasticity of human capital in Northeast China.

Take logarithm on both sides of the above equations, and the specific model after taking logarithm can be expressed as Equation 3.


(3) ln⁢Y=A+α⁢ln⁢L+β⁢ln⁢K+γ⁢ln⁢H

Among them, *ln Y* is the predicted variable, while *ln K*, *ln L*, and *ln H* are explanatory variables. Finally, the regression analysis is conducted on the relationship between labor force and regional economic growth, material capital and regional economic growth, and human capital and regional economic growth in Northeast China by the least square method.

The data of Northeast China from 2000 to 2020 are selected for empirical analysis to study the individual impact of labor force, material capital, and human capital on regional economic growth in Northeast China. Furthermore, Chen (2019) showed that enterprises tended to hire candidates at the suitable technical level. Moreover, it is necessary to select several corresponding measure indexes, including regional economic growth, human capital stock, material capital investment, and labor stock. The data of per capita GDP (Gross Domestic Product) in Northeast China over the years are selected as an index to measure regional economic development. Besides, the human capital stock in Northeast China is evaluated by the product of the year-end proportion of the number of people with higher education to the individual operators and the average years of education. Moreover, the year-end number of individual operators in Northeast China is taken as the measure index of labor stock. Finally, Wang (2019) found that the whole society fixed asset investment is used as the measure index of material capital investment in Northeast China.

### Research on the Psychological Quality of New Entrepreneurs

#### Questionnaire Design

Gao et al. (2020) suggested that the “Questionnaire on Entrepreneurial Psychological Capital and New Venture Performance” for empirical research consists of three sub-questionnaires based on the questionnaire design of Wu and Song (2019). They divided the questionnaire into “basic information of selected entrepreneurs,” “sub-questionnaire for entrepreneurial psychological capital,” and “sub-questionnaire for new venture performance” considering the four gratification factors in entrepreneurial groups, namely trust, profit, learning, and social. Besides, there are only selection questions in all the three sub-questionnaires printed on both sides of a piece of paper, with a total of two pages of folded paper.

Ren and Ji (2019) indicated that the sub-questionnaire for entrepreneurial psychological capital adopts the Chinese version of the Psychological Capital Questionnaire developed and verified by Luthans F, Youssef C M, and Avolio B J, as shown in Table 1. The sub-questionnaire includes the 24 items (No. 13, No. 20, and No. 23 are reverse scoring items) as follows: No. 1 to No. 6 test entrepreneurs’ hope level, No. 7 to No. 12 test entrepreneurs’ self-efficacy level, No. 13 to No. 18 test entrepreneurs’ resilience level, and No. 19 to No. 24 test entrepreneurs’ optimism level. The sub-questionnaire here adopts the scoring method of the seven-point Likert scale.

**TABLE 1:** Items of the sub-scale measuring psychological capital of new entrepreneurs.

**Measurement dimension**	**Hope**						

Psychological capital measurement items for new entrepreneurs	If I encounter difficulties at work, I can find ways to solve them by myself.	I am full of energy for work.	I always have many solutions to any problems.	At present, I think I have made great achievements in my work.	I can think of many ways to achieve the work goals I set.	At present, I am achieving the work goals I set for myself.	So far I have achieved my goals.
Number	H1	H2	H3	H4	H5	H6	H7

**Measurement dimension**	**Self-efficacy**						
Psychological capital measurement items for new entrepreneurs	I believe I can analyze long-term problems and find solutions.	I feel very confident when leaders state things within their scope of work at a meeting.	I believe I can contribute to the discussion of the company’s strategy.	Within the scope of my work, I believe I can help set goals.	I believe I can contact suppliers and customers and discuss problems.	I believe I can present information to a group of colleagues.	I believe I can state my thoughts on any occasion.
Number	E1	E2	E3	E4	E5	E6	E7

The score of each item is added up as the score of each dimension of new entrepreneurs’ psychological capital, and the sum of each dimensional score represents the total score of entrepreneurs’ psychological capital. The sub-questionnaire for new venture performance adopts the new venture performance subjective measurement scale, which includes four items, namely entrepreneurs’ evaluation of the importance and satisfaction of net profit growth, sales growth, employee growth, and market share growth.

The sub-scale adopts the scoring method of the seven-point Likert scale. The Likert scale was improved by American social psychologist Likert based on the original summated rating scales in 1932 (Batarseh, 2020). This scale consists of a group of questions or statements related to a topic. By calculating the total scores of each question of the scale, researchers can understand people’s comprehensive attitude or view on the survey topic. There are many forms of Likert scales, among which the five-point scale with five items is most common. In addition, there are usually seven-point, nine-point, and four-point scales. The answers to questions of the scale range from one extreme attitude to another, such as “very likely” to “impossible at all,” or “very agree” to “very disagree.” Compared with the binary question that only provides two answer options, the Likert items can more accurately feedback the respondents’ attitude toward the question, to collect more accurate data. Meanwhile, most of the statistical methods are particularly appropriate for scales, such as reliability analysis, validity analysis, and exploratory factor analysis, which can collect abundant and accurate data.

The scores of new venture performance are the sum of the scores of four items which involve the subjective evaluation scores of entrepreneurs on the importance and satisfaction.

#### Questionnaire Distribution and Collection

Eight emerging enterprises in Jilin Province that have been established for no more than 4 years in the List of the Most Growing Emerging Enterprises in 2020 published by the *Chinese Entrepreneur* are selected as research samples. A total of 200 questionnaires are distributed by convenient sampling and random sampling in these eight enterprises from June 1, 2020 to December 1, 2020. Finally, 168 valid questionnaires are collected despite the 19 invalid questionnaires with inattentive answers or deception or more than 6 years among 187 returned questionnaires.

#### Data Analysis Method

This chapter intends to use IBM SPSS 26.0 for statistical analysis of the data obtained to test the proposed hypotheses. The questionnaire “Entrepreneur Psychological Capital and New Venture Performance Questionnaire” used here for large sample survey includes two sub-scales “Entrepreneur Psychological Capital Scale” and “New Venture Performance Scale.” Moreover, reliability analysis and validity analysis are conducted on the questionnaire. The reliability refers to the stability or consistency of the test or scale results. The higher the reliability, the smaller the standard error of the measurement. There are generally three common reliability indicators, namely stability, equivalence, and internal consistency. The validity of measurement denotes the correctness of measurement. The confirmatory factor analysis is performed on the two sub-scales.

The data analysis methods for the data of the questionnaire survey include the following:

Qin (2021) suggested that descriptive statistics are mainly for the basic situation of sample entrepreneurs and sample enterprises, and the main indicators include frequency, percentage, effective percentage, and cumulative percentage.

Confirmatory factor analysis and reliability analysis: confirmatory factor analysis is used to test the construct validity and the data fitting ability of the scale. The reliability of the scale is presented as values obtained by the reliability analysis.

Validity analysis: Kaiser–Meyer–Olkin (KMO) test statistic is used to measure scale validity, which is an indicator to compare the simple correlation coefficient and partial correlation coefficient between the variables. KMO is mainly used in multivariate statistical factor analysis. The value of KMO statistics is between 0 and 1.

When the sum of squares of simple correlation coefficients between all variables is far greater than the sum of squares of partial correlation coefficients, the KMO value is closer to 1. This means that the correlation between the variables is strong, and the original variables are more suitable for factor analysis. When the sum of squares of simple correlation coefficients between all variables is close to 0, the KMO value is closer to 0, which indicates that the correlation between the variables is weak, and the original variables are not suitable for cooperative factor analysis.

**FIGURE 3:**
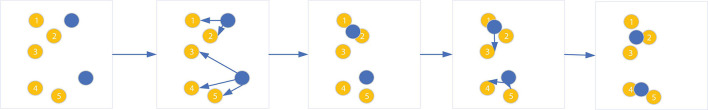
Means clustering analysis method.

Comparison of mean: the k-means cluster analysis method is adopted here to test whether there are significant differences in entrepreneurial psychological capital at different levels in the performance of new ventures. The k-means cluster analysis is a data mining method, mainly to calculate data aggregation, which achieves aggregation primarily by constantly taking the nearest mean of the seed point. Figure 3 illustrates the algorithm flow in which entrepreneur psychological capital is divided into three groups including high level, medium level, and low level for single-factor variance analysis.

Regression analysis: multiple regression analysis is more commonly used in empirical research. One of the main purposes is to understand the relationship between independent variables and dependent variables, and the direction and degree of influence of independent variables on dependent variables. In terms of specific methods, regression analysis mainly includes enter, stepwise, remove, backward, and forward.

### Psychological Capital Model of New Entrepreneurs Based on PCI Model

**FIGURE 4:**
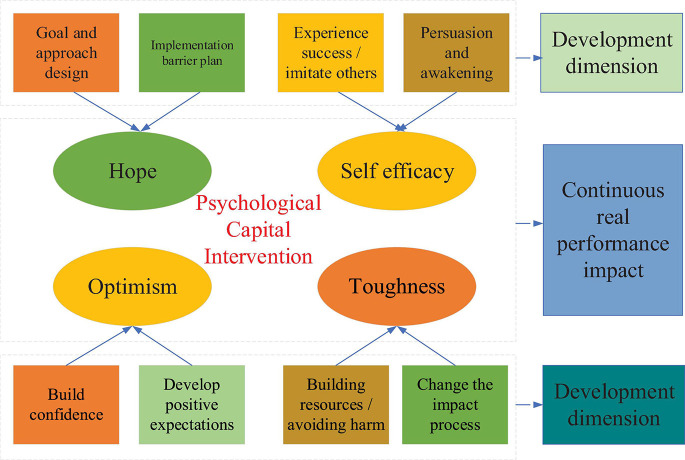
PCI model.

Patnaik et al. (2021) pointed out that the PCI model is a famous model proposed by Luthans based on positive psychology and positive organizational behavior to develop individual psychological capital. The PCI model is shown in Figure 4.

**FIGURE 5:**
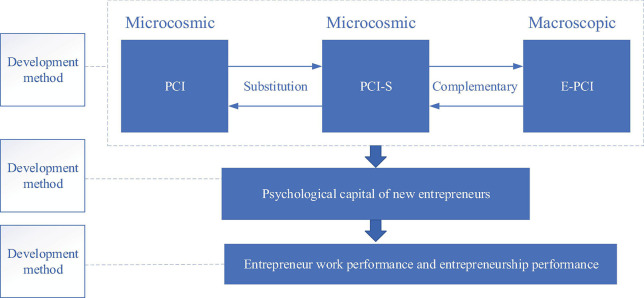
E-PCI-S model for developing entrepreneurs’ psychological capital.

Due to the limitations of the PCI model and the particularity of the psychology and behavior of new entrepreneurs, a multilevel and multiway external environment psychological capital intervention instead of external environment PCI (E-PCI-S) model for developing entrepreneurs’ psychological capital. This model combines macro-development and micro-development, direct development and indirect development, and active development and passive development, as shown in Figure 5.

The E-PCI-S model consists of three sub-models. Firstly, the PCI model is the basis of the E-PCI-S model, which means that entrepreneurs can develop psychological capital through the external treatment of PCI. Secondly, E represents the external environment, and the E-PCI model can develop entrepreneurs’ psychological capital by optimizing the external environment. Thirdly, Song et al. (2019) stated that the PCI-S model referring to PCI and substitution indicates the indirect or circuitous development of psychological capital of entrepreneurs through the investment of other capital (except psychological capital), as shown in Table 2. Among the three sub-models, the E-PCI model and PCI-S model belong to the macro-development, while the PCI model belongs to the micro-development, and the development process is initiated and indirect.

**TABLE 2:** Sub-models of the E-PCI-S model.

**Name**	**PCI**	**PCI-S**	**E-PCI**
Development subject	External experts	Entrepreneurs	The carrier of external environment (government departments or the social environment)
Development object	Entrepreneurs who tend to focus and are open to external therapeutic development.	Entrepreneurs with strong independence and autonomy, high self-efficacy, low concentration and unwillingness to receive external treatment.	All entrepreneurs.
Development method	Short-term or high-focus face-to-face or network-based micro-interventions.	PCI and substitution, such as human capital investment.	Optimizing the external environment.
Development level	Microcosmic level	Microcosmic level	Macroscopic level
Development effect	The effectiveness is short, and duration and recurrence remain to be tested.	Short-term or long-term effect, and duration to be tested.	Long-term effect positively changing with the external environment.

As for the development subject, the E-PCI-S model includes not only external experts, but also entrepreneurs themselves and environmental carriers. In terms of development objects, the E-PCI-S model is applicable to those entrepreneurs easy to concentrate on and willing to accept external treatment development, and also those entrepreneurs with strong independence and autonomy, high self-efficacy, low concentration, and unwillingness to receive external treatment. On the development dimension, the E-PCI-S model includes not only four dimensions of the PCI model, but also other potential or future psychological capital dimensions, such as subjective well-being and emotional intelligence. Moreover, the E-PCI-S model pays attention to short-term and high-focus development methods such as face-to-face contact development or network-based development proposed by Yuan and Wu (2020) and PCI substitution such as human capital investment by Collin and Weil (2020). It also attaches great importance to a more macro- and gradual development method such as external environment optimization. The development effect of the E-PCI-S model remains to be tested. However, the E-PCI-S model is an expansion of the PCI model including the external environment and a new method. This part mainly expounds on the theoretical basis of the model from the influence of the external environment on psychological capital and the relationship between different forms of entrepreneurial capital.

## Results and Discussion

### Empirical Analysis of Human Capital and Economic Growth in Northeast China

**FIGURE 6:**
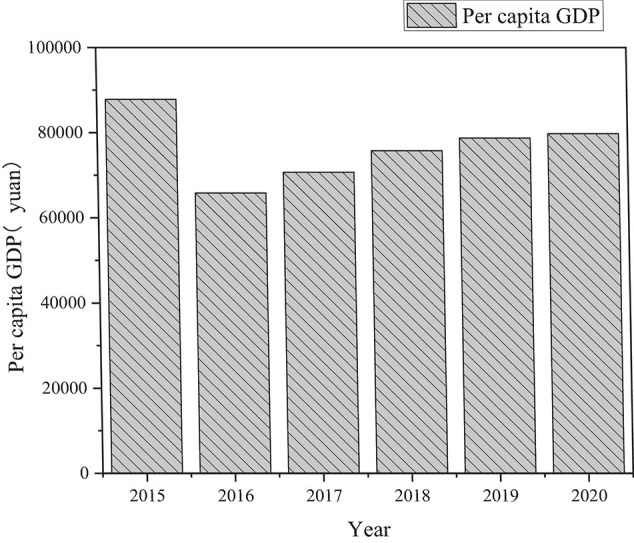
Statistics of per capita GDP in Shenyang from 2015 to 2020.

**FIGURE 7:**
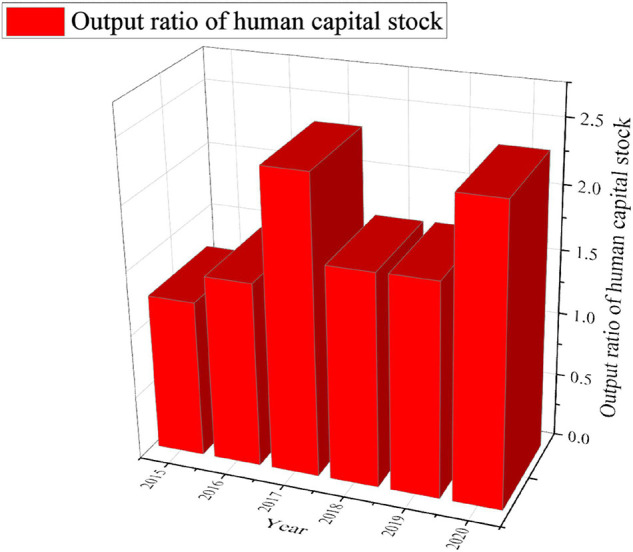
Output ratio of human capital stock in Shenyang from 2015 to 2020.

Taking Shenyang in eastern China as an example, the impact of labor, physical capital, and human capital on regional economic growth from 2015 to 2020 is analyzed. The specific results are shown in Figures 6, 7.

Figure 7 shows that the output ratio of human capital stock in Shenyang is basically stable between 1 and 3. The slow growth rate of human capital stock is one of the important factors restricting economic growth. In other words, economic growth in recent years is mainly driven by material capital investment, while the output efficiency of human capital stock is getting continuously lower. Therefore, it is an effective way for stimulating economic growth to promote the increase of human capital stock and improve the output efficiency of human capital.

In Table 3, *K* represents the material capital, *H* is the human capital, *L* denotes the labor force, and *A* shows the technical level. According to the above regression results, the variable coefficients are under 1% using the *t*-test significance level. Table 3 reveals that the elastic coefficient of labor *ln* L is 0.538595, while the elasticity coefficient of physical capital *ln K* is 0.486279. Besides, the elasticity coefficient of human capital *ln H* in is 0.150902, indicating that with other variables unchanged, the per capita GDP in Shenyang increases by 0.150902% for every 1% growth of human capital. Therefore, although human capital has a positive effect on the economic development of Northeast China, the impact of human capital is significantly lower than that of material capital and labor forth. That is to say, today Northeast China mainly relies on labor and material capital to drive its economic growth.

**TABLE 3:** Unit root test of variables.

**Original sequence**	***ln Y***	***ln H***	***ln L***	***ln K***
Numerical value	4.123421 (−2.908732)	−1.32412 (−2.987621)	0.42341 (−2.987633)	2.352311 (−3.098234)
Conclusion	Unstable	Unstable	Unstable	Unstable
First-order difference	D (*ln Y*)	D (*ln Y*)	D (*ln Y*)	D (*ln Y*)
Numerical value	−1.543232 (−3.572832)	−1.724422 (−3.230131)	−2.342432 (−3.482421)	−2.342211 (−3.452232)
Conclusion	Unstable	Unstable	Unstable	Unstable
Second-order difference	DD (*ln Y*)	DD (*ln Y*)	DD (*ln Y*)	DD (*ln Y*)
Numerical value	−4.232423 (−3.232421)	−5.340922 (−3.232345)	−5.348242 (−3.032342)	−5.334824 (−3.264342)
Conclusion	Stable	Stable	Stable	Stable

### Sample Analysis of New Entrepreneurs

**FIGURE 8:**
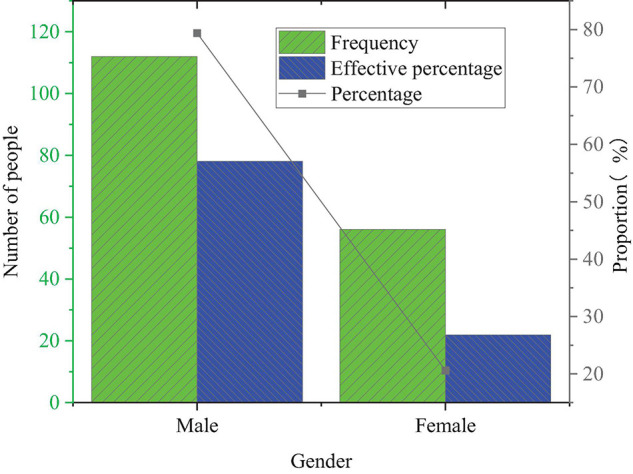
Descriptive statistics of the gender of the sample entrepreneurs.

Descriptive statistics of the gender of the selected entrepreneurs are shown in Figure 8.

In Figure 8, the sample entrepreneurs for 168 questionnaires contain 112 men and 56 women, and the proportion of men is much higher than that of women. This phenomenon may be caused by the limited economic development level and the undeveloped entrepreneurial culture atmosphere in Northeast China, which leads to the fact that men are mainly engaged in entrepreneurial activities.

**FIGURE 9:**
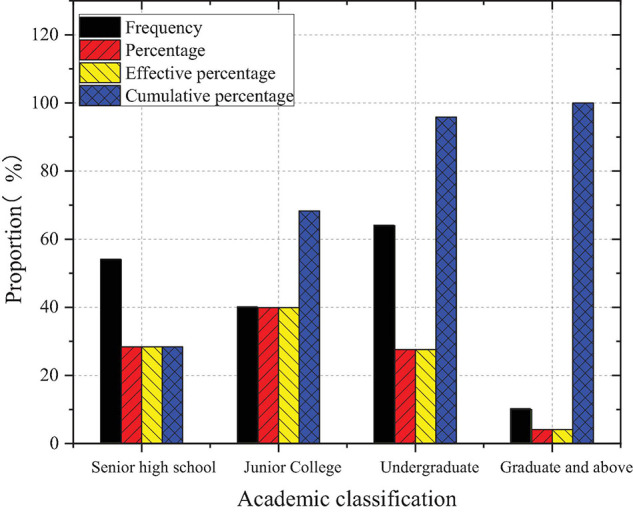
Descriptive statistics of sample entrepreneurs’ educational levels.

Figure 9 describes the descriptive statistics of sample entrepreneurs’ educational levels.

According to Figure 9, the majority of the selected entrepreneurs have a college degree, and less than 30% have a high school (or secondary) diploma or below, which is far less than 50% reported in the *Global Entrepreneurship Monitor 2020 China Report*. This proves the rationality of the classification of “education level” in this study from another aspect. It shows that more new entrepreneurs are willing to receive higher education.

Reliability coefficient Cronbach *α* is a statistic, the average value of split-half reliability coefficient obtained by all possible item division methods. It is the most commonly used reliability measurement method. The analysis results of Cronbach *α* are presented in Table 4.

**TABLE 4:** Reliability coefficient of the two sub-questionnaires.

**Dimension**	**Hope**	**Self-efficacy**	**Resilience**	**Optimism**	**Sub-questionnaire for entrepreneurial psychological capital**	**Sub-questionnaire for new venture performance**
Number of items	5	4	6	2	19	5
Cronbach *α*	0.823	0.789	0.812	0.783	0.925	0.802

The Cronbach *α* of the sub-questionnaire for entrepreneurial psychological capital reaches an ideal value of 0.918. Besides, the Cronbach *α* values of resilience dimension, hope dimension, and the sub-questionnaire for new venture performance are all above 0.80, which is good. The Cronbach *α* of rest scales is above 0.75. Reliability analysis shows that the adopted questionnaire meets the requirement of research measurement.

The *x*^2^/df chi-square, NFI (Normed Fit Index), CFI (Comparative Fit Index), and RMSEA (Root Mean Square Error of Approximation) are used as indicators to test the validity of the measurement scale. The KMO value of the validity result is 0.89. Table 5 elucidates the confirmatory factor analysis results of the Entrepreneur Psychological Capital Scale and New Venture Performance Scale.

**TABLE 5:** Confirmatory factor analysis results of two sub-scales.

	**H (Hope)**	**E (Self-efficiency)**
*x*^2^/df	1.39	1.68
NFI	0.93	0.91
CFI	0.94	0.94
RMSEA	0.043	0.055

The RMSEA value of E in Table 5 is 0.05 (0.1 > 0.055 > 0.05), which indicates an acceptable fitting, belonging to the good range. Confirmatory factor analysis shows that the two sub-scales both have a good fitting ability to the data.

### Analysis of Psychological Capital Development of New Entrepreneurs in Northeast China Based on PCI Model

**FIGURE 10:**
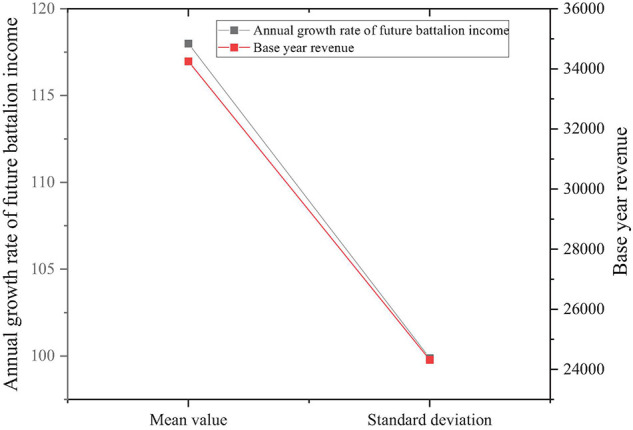
Base year revenue and average annual growth rate of future revenue.

Based on the data of per capita GDP and human capital stock in Northeast China over the years, the average annual growth value of future revenue is shown in Figure 10.

According to Figure 9, new entrepreneurs must spend 378.75 h on average to increase their psychological capital by 3.7875 on average with an average cost of 11.58975 million CNY to achieve an average annual revenue growth rate of 117.00% in the future. From the proportion of cost to future revenue (1,158.975/33,543.60 ≈ 0.035), it seems reasonable to develop new entrepreneurs’ psychological capital based on the PCI model. However, there will be problems when further examining the cost-benefit conditions, the life cycle and the environmental uncertainty of selected enterprises and the quantity of emerging enterprises, or under the simulation of the real world by adding some “moderating variables.” For example, the eight emerging enterprises spend more than 100 million CNY on psychological capital development to obtain the expected revenue growth. If the number of enterprises is further expanded, it will be essential to consider the macro-economic issue of the economic benefit of the individual cost of psychological capital development of many emerging enterprises. Obviously, there are some limitations in developing the psychological capital of new entrepreneurs based on the PCI model.

## Conclusion

The former planned economic system greatly affecting Northeast China is not conducive to independent production and free competition of enterprises, lying a certain impediment to the appointment and employment of talents. The innovative analytic model is proposed to estimate the cost of developing new entrepreneurs’ psychological capital based on the PCI model under samples of eight emerging enterprises in Northeast China. The discussion results show that the PCI model has insurmountable inherent defects in developing the psychological capital of new entrepreneurs, such as the possible cost-benefit asymmetry, cash flow risk, uncertainty of investment results, and the non-scale economy of development. As an alternative method, the E-PCI-S model has not only a desirable and solid empirical basis but also a huge operating space under the premise of optimizing the macro-entrepreneurial environment.

Some progress of research perspective, research content, and research methods has been achieved in this study. However, there are some limitations with theoretical and practical significance for the further study of the structure and the cultivation of entrepreneurial psychological quality and the enhancement of entrepreneurial ability. For example, the sampling range of empirical research is relatively narrow, which may affect the external validity of empirical research conclusions. It is expected that more mediating variables and moderating variables can be introduced in the following research to determine the conditions and scope of entrepreneurial psychological capital efficacy and to open the “black box” of entrepreneurial psychological capital efficacy.

## Data Availability Statement

The raw data supporting the conclusions of this article will be made available by the authors, without undue reservation.

## Ethics Statement

The studies involving human participants were reviewed and approved by Harbin University of Science and Technology Ethics Committee. The patients/participants provided their written informed consent to participate in this study. Written informed consent was obtained from the individual(s) for the publication of any potentially identifiable images or data included in this article.

## Author Contributions

All authors listed have made a substantial, direct, and intellectual contribution to the work, and approved it for publication.

## Conflict of Interest

The authors declare that the research was conducted in the absence of any commercial or financial relationships that could be construed as a potential conflict of interest.

## Publisher’s Note

All claims expressed in this article are solely those of the authors and do not necessarily represent those of their affiliated organizations, or those of the publisher, the editors and the reviewers. Any product that may be evaluated in this article, or claim that may be made by its manufacturer, is not guaranteed or endorsed by the publisher.
